# Cognitive function is associated with risk of conversion to secondary progressive multiple sclerosis: A cross-sectional study

**DOI:** 10.1371/journal.pone.0344558

**Published:** 2026-03-20

**Authors:** Omid Mirmosayyeb, Ida Mohammadi, Shahryar Rajai Firouzabadi, Saeed Vaheb, Mohammad Yazdan Panah, Tom A. Fuchs, Bianca Weinstock-Guttman, Vahid Shaygannejad

**Affiliations:** 1 Department of Neurology, Jacobs Comprehensive MS Treatment and Research Center, Jacobs School of Medicine and Biomedical Sciences, University at Buffalo, State University of New York, Buffalo, New York, United States of America; 2 Student Research Committee, School of Medicine, Shahid Beheshti University of Medical Sciences, Tehran, Iran; 3 Isfahan Neurosciences Research Center, Isfahan University of Medical Sciences, Isfahan, Iran; 4 Department of Anatomy and Neuroscience, Amsterdam University Medical Center, Amsterdam, The Netherlands; 5 Department of Neurology, School of Medicine, Isfahan University of Medical Sciences, Isfahan, Iran; University College London, UNITED KINGDOM OF GREAT BRITAIN AND NORTHERN IRELAND

## Abstract

**Background:**

Predicting conversion from relapsing-remitting multiple sclerosis (RRMS) to secondary progressive multiple sclerosis (SPMS) is critical for managing people with multiple sclerosis (PwMS). Cognitive function has been linked to disease progression in RRMS, yet its association with conversion to SPMS remains underexplored. This study aimed to assess this relationship using the newly developed DAAE score to calculate the risk of conversion to SPMS.

**Methods:**

The risk of conversion over five years was assessed using the DAAE score (0–12), and the PwMS were categorized into very-low-to-low and medium-to-high risk groups. Cognitive function was assessed using the Symbol Digit Modalities Test (SDMT), Brief Visuospatial Memory Test-Revised (BVMT-R), Paced Auditory Serial Addition Test (PASAT), and California Verbal Learning Test-Second Edition (CVLT-II). Pairwise correlations and hierarchical linear and logistic regression analyses were performed to examine the relationship between cognition and conversion risk.

**Results:**

A total of 217 PwMS were included (very low to low = 185, medium to high = 32). The DAAE score was moderately correlated with SDMT and BVMT-R and weakly correlated with PASAT and CVLT-II. Multivariable linear regressions found SDMT (beta = −0.125, 95%CI: −0.155, −0.096, p-value < 0.001), BVMT-R (beta = −0.157, 95%CI: −0.200, −0.114, p-value < 0.001), PASAT (beta = −0.084, 95%CI: −0.111, −0.057, p-value < 0.001), and CVLT-II (beta = −0.088, 95%CI: −0.125, −0.050, p-value < 0.001) were independently associated with the risk of conversion. Among these, SDMT (beta = −0.093, 95% CI: −0.133 to −0.054, p-value < 0.001) was the most robust predictor of conversion risk.

**Conclusion:**

In conclusion, low cognitive performance across the BICAMS battery and the PASAT, with the SDMT as the most robust predictor, is associated with an increased risk of conversion from RRMS to SPMS. By demonstrating this association through a machine-learning framework, the present study supports the integration of standardized neuropsychological assessments into routine clinical practice as tools for monitoring conversion risk in PwMS, beyond conventional clinical measures.

## 1. Introduction

Multiple sclerosis (MS) is a chronic inflammatory demyelinating disease of the central nervous system (CNS) that primarily affects young adults [1]. The disease manifests with heterogeneous courses: a minority of people with MS (PwMS) experience a gradual decline in neurological function, termed primary progressive MS (PPMS), whereas most experience a relapsing-remitting disease course (RRMS), characterized by acute exacerbations followed by partial or complete recovery, interspersed with periods of relative clinical stability [2,3]. The natural progression of RRMS often involves a transition to secondary progressive multiple sclerosis (SPMS), marked by a gradual increase in irreversible disability that occurs independently of relapses [4]. Historically, up to 80% of RRMS patients would eventually convert to SPMS, but earlier diagnosis and improved treatments have reduced 10-, 15-, and 20-year conversion rates to 2%, 9%, and 27%, respectively [5].

Since cognitive impairment affects 40–70% of PwMS [6], routinely administered neuropsychological assessments are critical to the comprehensive evaluation of PwMS [7]. There is currently no consensus on the most suitable neuropsychological battery for assessing cognitive impairment in MS, and several instruments are used in both clinical practice and research. Commonly applied batteries include the Brief Repeatable Battery of Neuropsychological Tests (BRB-N), the Minimal Assessment of Cognitive Function in MS (MACFIMS), and their derivatives, each evaluating partially overlapping cognitive domains [8]. The BRB-N is widely considered a relatively comprehensive yet time-efficient battery that provides a broader characterization of cognitive functioning in people with MS. Due to its wider domain coverage, it has been described as more suitable for identifying cognitive change over time, particularly in longitudinal research settings. In contrast, the Brief International Cognitive Assessment for Multiple Sclerosis (BICAMS), derived from MACFIMS, was specifically developed to facilitate standardized cognitive screening in everyday clinical environments, and has been proposed as a pragmatic tool to support broader implementation beyond specialized neuropsychological settings [9,10]. Therefore, while the BRB-N may offer a more comprehensive evaluation of cognitive change, BICAMS is a pragmatic, scalable tool that is particularly suitable for incorporation into routine patient care. The BICAMS, which incorporates the Symbol Digit Modalities Test (SDMT) for processing speed, the California Verbal Learning Test-II (CVLT-II) for verbal memory, and the Brief Visuospatial Memory Test-Revised (BVMT-R) for visuospatial memory [11], as well as the Paced Auditory Serial Addition Test (PASAT) in some settings [12]. Neuroimaging studies have consistently linked cognitive impairment in PwMS to structural and functional brain changes. Grey matter atrophy in cortical and hippocampal regions correlates with deficits in memory encoding and retrieval, as well as visuospatial memory [13]. Additionally, cortical lesions and corpus callosum atrophy have been associated with slower processing speed, reduced attention, and poorer executive function [14]. In addition, neuroimaging studies have shown that MRI indices, such as thalamic volume [15] and microstructural changes in normal-appearing brain matter [16], are associated with impairments in attention, processing speed, executive function, and memory [17,18].

Neuropsychological studies across clinical subtypes show more pronounced cognitive deficits in progressive forms of MS compared to RRMS. Denney et al. reported greater impairment in SPMS than in PPMS [19]. Similarly, Katsari et al. identified deficits in episodic memory and executive functions, specifically in SPMS patients [20]. Additionally, Ntoskou et al. found that cognitive performance in PwMS deteriorates significantly as they transition from RRMS to SPMS [21]. Importantly, cognitive impairment can occur even in the absence of physical disability, suggesting that cognitive decline may serve as an early indicator of disease progression and conversion to SPMS [22]. A systematic review of biomarkers for conversion to SPMS identified the SDMT as one of the most promising predictors [5]. The SDMT measures cognitive processing speed and is highly sensitive to cognitive impairment in PwMS, making it an effective screening tool [23]. Notably, among a variety of neuropsychological and language performance measures, including PASAT, the SDMT has displayed the greatest effect size in distinguishing between RRMS and SPMS [21,24]. Furthermore, baseline SDMT performance predicts long-term progression in SPMS, with lower scores associated with an increased risk of reaching wheelchair dependence (EDSS ≥7) over follow-up periods up to 5 years, highlighting its value in identifying PwMS at higher risk of disability progression [25]. However, evidence for other neuropsychological measures remained limited.

To better understand cognitive decline in PwMS at high risk of conversion from RRMS to SPMS, the disease’s underlying mechanisms must be discussed. Disease activity and progression are key components of MS, offering insights into RRMS and SPMS [26]. In RRMS, disease activity, marked by relapses and lesion changes on CNS imaging, reflects focal inflammatory demyelination [27]. SPMS, however, is characterized by progression, involving worsening neurological dysfunction linked to plaque expansion and neurodegeneration [28]. Previously viewed as distinct processes in RRMS and SPMS, inflammation and neurodegeneration are now understood as a continuum [3]. Recent studies have revealed that “silent progression” in RRMS, where disability advances without relapses [29], is associated with cortical atrophy [30], suggesting that the process of neurodegeneration starts earlier than previously thought. Changes in cognition can also result from this neurodegeneration, as annual cognitive decline has been shown to correlate with the rate of annual deep gray matter atrophy in the conversion from RRMS [31].

The role of cognitive decline as an early marker of disease progression has important prognostic implications. Importantly, it is relevant to disease-modifying therapies (DMTs). While DMTs are highly effective in reducing relapse activity and slowing disability accumulation in RRMS, their benefits in non-relapsing SPMS are limited. As a result, anticipating and preventing conversion has become a major research priority [5]. A recently developed tool, the DAAE (disease duration, age at onset, age, Expanded Disability Status Scale; EDSS), has shown strong predictive accuracy for estimating the 5-year risk of conversion from RRMS to SPMS and has outperformed machine learning models in validation studies across international datasets [32]. Given the novelty of the DAAE score and the inconclusive evidence regarding the role of different cognitive domains in conversion, we aimed to investigate whether information processing speed, visuospatial memory, and verbal learning and memory are associated with the risk of SPMS conversion, as measured by the DAAE score.

## 2. Methods

This cross-sectional study was conducted at Kashani Hospital in Isfahan, Iran, between September 2023 and September 2024. It adhered to the ethical principles of the Declaration of Helsinki and received approval from the Ethics Committee of Isfahan University of Medical Sciences (registration code: IR.ARI.MUI.REC.1401.061). Written informed consent was obtained from all PwMS before enrollment.

### 2.1. Participants

Adults (≥18 years) diagnosed with RRMS according to the revised McDonald’s criteria 2017 [33] and with at least one year since diagnosis were eligible. Exclusion criteria included other neurological or chronic diseases (e.g., gastrointestinal, cardiac, hepatic, renal, or respiratory disorders), pregnancy or breastfeeding, and psychiatric disorders other than generalized anxiety disorder (GAD), bipolar disorder (BPD), major depressive disorder (MDD), and obsessive-compulsive disorder (OCD). An expert psychiatrist confirmed the diagnoses of these psychiatric conditions. No upper age limit was applied to include the full spectrum of adult RRMS patients. There were no restrictions regarding the severity of MS or the type of treatment. Educational background was recorded as the total number of years of formal education, starting from the first year of primary school through the final completed degree. In our sample, a median of 16 years corresponds approximately to completion of a bachelor’s degree in the Iranian educational system.

### 2.2. Study variables and endpoints

Demographic data (age, sex, years of education) and clinical data (age at disease onset, disease duration, type of DMT, and EDSS [34]) were collected and assessed by a neurologist. Cognitive function was assessed using the PASAT and the BICAMS, which includes the SDMT, CVLT-II, and BVMT-R [11]. The DAAE score was calculated for each patient [32] based on demographic and clinical variables. A detailed description of each assessment tool used in this study is provided below.

#### 2.2.1. Symbol digit modalities test.

The SDMT is a measure of sustained attention and cognitive processing speed. Participants are shown a stimulus page displaying a key with nine number-symbol pairs. Within 90 seconds, participants verbally identify the correct number associated with each target symbol. The performance is scored based on the total number of correct responses, with possible scores ranging from 0 to 110 [35,36]. A validated Persian translation of the SDMT was used [37].

#### 2.2.2. California verbal learning test- second edition.

The CVLT-II is an auditory and verbal learning and memory test and evaluates immediate recall. A validated Persian version [37] was used to assess memory using a 16-item word list divided into four semantic categories (vegetables, animals, modes of travel, and furniture). Participants listened to the list and recalled the words over five trials. The total recall score, ranging from 0 to 80, was calculated by summing the number of words recalled across all trials [38].

#### 2.2.3. Brief visuospatial memory test-revised.

The BVMT-R assesses visuospatial memory through immediate recall. Participants are shown six abstract designs for 10 seconds; after the display is removed, they are asked to replicate the designs on paper. Each design is scored from 0 to 2 based on accuracy and placement, resulting in a total score ranging from 0 to 12 per trial. The test includes three learning trials, with the primary outcome being the cumulative score across all trials, ranging from 0 to 36 [11].

#### 2.2.4. Paced auditory serial addition test.

The PASAT assesses auditory information processing speed (IPS), mental flexibility, and calculation ability. Originally developed for traumatic brain injury [39], it was later adapted for use in PwMS [40]. During the test, single digits were presented via audiotape every 3 seconds, and participants added each new digit to the preceding one. The primary outcome was the number of correct responses (maximum score: 60) [41]. A validated Persian version of the test was used in this study [37]. Although the PASAT was evaluated by the BICAMS expert committee and not retained in the final recommended battery due to concerns regarding reliability and patient burden [42], we included it in the present study. Our rationale was to complement the BICAMS sub-tests by assessing auditory IPS and working memory, domains that are highly relevant in MS, and are still a robust marker in certain MS cognitive assessments [43–45].

#### 2.2.5. Risk of conversion to SPMS.

The DAAE score estimates the risk of conversion from RRMS to SPMS over five years. It is calculated by assigning a score in relation to disease duration (up to 3 points), age at the onset of disease (up to 1 point), age (up to 2 points), and EDSS (up to 6 points), and then summing up the scores to reach a total ranging from 0 to 12.

#### 2.2.6. Conversion risk status.

This study is cross-sectional. Therefore, instead of longitudinal follow-up, the risk of transition to SPMS was estimated using the DAAE score. The risk of conversion was defined as the sum of the DAAE scores. Scores from 0–2, 3–7, 8–9, and> nine signify very low, low, medium, and high risk of conversion, respectively [32]. Due to the similar observed conversions in the very low and low groups in the original DAAE study [32] alongside the limited number of PwMS in the high risk of conversion group, conversion risk status was classified into very low to low versus moderate to high (0–7 versus 8–12) risk.

### 2.3. Statistical analysis

All analyses were conducted using Stata version 18 [46]. For continuous variables, normality of the distribution was checked using the Shapiro-Wilk test they were presented as means (standard deviations) or medians (Q1-Q3) based on the normal distribution. Ordinal variables (e.g., education) were treated as continuous for descriptive analyses but as ordinal in regression analyses and were reported accordingly. Nominal variables are reported as frequencies or percentages. When comparing continuous variables between PwMS with very-low-to-low risk (DAAE ≤7) and medium-to-high risk (DAAE >7), a two-sample t-test was used in case the variable was normally distributed, or a Mann–Whitney test was conducted in case of non-normal distribution. For categorical variables, the Pearson’s chi-squared test was used. In case of heterogeneity of variances, the t-test would be adjusted accordingly.

To investigate the association between cognitive domains and the risk of conversion, pairwise correlations were conducted using Spearman correlation coefficients (⍴) due to the non-normal distribution of DAAE scores. Weak, moderate, and strong correlations were defined as ⍴ < 0.4, 0.4 ≤ ⍴ < 0.7, and ⍴ ≥ 0.7, respectively [47]. Furthermore, a set of hierarchical linear and logistic regressions was further developed to identify independent predictors of the risk of conversion and conversion risk status, respectively. Step one comprised covariates (sex, education, presence or absence of psychiatric comorbidities, and type of DMT), and step two comprised one cognitive test (PASAT, SDMT, CVLT-II, or BVMT-R). Finally, another set of hierarchical linear and logistic regressions, including all cognitive tests in step two, was conducted to assess which cognitive domains are most associated with the risk of conversion. The results of the regression models are reported as unstandardized coefficients, standardized beta coefficients (beta), or odds ratios (OR), with their respective 95% confidence intervals (95% CI). A p-value < 0.05 was considered significant.

All regression models were checked for compliance with statistical assumptions. Specifically, we examined the normality of the residuals using the Shapiro–Wilk test, homogeneity of variance using Levene’s test, and multicollinearity using variance inflation factors (VIFs). The diagnostic results confirmed that these assumptions were adequately satisfied for the presented analyses.

#### 2.3.1. Effect sizes and post hoc power analysis.

For hierarchical linear models, we computed incremental effect sizes as the change in explained variance (ΔR²) when adding each cognitive test, and derived Cohen’s f²Δ using the following formulas:


$$\Delta {𝐑}^{\text{2}}\;=\;{\text{R}}^{\text{2}}\_\text{full }-\;{\text{R}}^{\text{2}}\_\text{reduced}$$

$${\text{f}}^{\text{2}}\Delta \;=\;\Delta {𝐑}^{\text{2}}\;/\;(\text{1}\;-\;{\text{R}}^{\text{2}}\_\text{full})$$

A post hoc sensitivity analysis using G*Power Software version 3.1.9.7 (G*Power-style; linear multiple regression, fixed model: R² increase; N = 217, α = 0.05) was conducted to quantify achieved power for the observed f²Δ. Furthermore, independence of errors was assumed, given the absence of identifiable clusters in the dataset and the fact that all PwMS came from the same geographic location. For logistic models, we report odds ratios (ORs) with 95% confidence intervals and pseudo-R²; power based on pseudo-R² is considered approximate.

#### 2.3.2. Secondary analysis using a Random Forest.

In addition to conventional regression analyses, secondary analyses were conducted to disentangle the association between cognitive performance and conversion risk while accounting for potential confounding between EDSS and cognitive dysfunction. For this purpose, a non-linear machine-learning framework was applied. This approach allowed us to assess whether the inclusion of cognitive test scores in a random forest classifier would enhance predictive performance and alter model behavior, providing a more robust evaluation of non-linear interactions and conditional dependencies than conventional statistical inference models [48].

We used stratified splitting as described previously [49] to split the dataset 80/20 into train and test subsets, and then we trained the random forest models using four distinct sets of inputs. The baseline model incorporated demographic variables and the type of disease-modifying therapy. Model 1 expanded upon the baseline by including four cognitive test scores. Model 2 combined the baseline features with EDSS, and model 3 incorporated all features from models 1 and 2. Model performance was assessed using the area under the ROC curve and the cut-point derived from the maximum value of Youden’s index derived from the training set, and hyperparameters were selected through five-fold cross-validation before retraining on the full training subset. For model 3, feature importance (mean decrease impurity) [50], partial dependence plots, and SHAP values were visualized to characterize the contribution of individual predictors across the training samples. Analyses were carried out using Scikit-learn [51] and SHAP packages within Python v3.10.

## 3. Results

### 3.1. Population characteristics

In total, 217 PwMS were included in our study. The mean age of the PwMS was 38.08 years (SD = 8.54) with a median age of onset of 28 years (Q1-Q3 = 22–34). Most of the PwMS (90.78%) were female and had a median of 16 years of education (Q1-Q3 = 12–16). Among them, psychiatric comorbidities were present in six cases with GAD, five with OCD, seven with MDD, and three with BPD. The PwMS had a median EDSS score of 2 (Q1-Q3 = 1–3) and a median disease duration of 9 years (Q1-Q3 = 5–13). In total, 47 (21.66%) of the PwMS received oral DMTs, while 81 (37.33%) received infusion DMTs and 89 (41.01%) received injectable therapies. Cognitive assessments revealed that the PwMS had average scores of 47.03 (SD = 11.12) on the SDMT, a mean CVLT-II total recall score of 48.38 (SD = 9.52), a median PASAT score of 45 (Q1-Q3 = 36–54), and a median BVMT-R score of 26 (Q1-Q3 = 19–30). The median DAAE was 4 (Q1-Q3 = 2–6). The majority of PwMS (85.3%) were categorized as having a very low to low risk of conversion, while 32 (14.7%) were categorized as having a medium-to-high risk of conversion. Table 1 compares the characteristics of the very-low-to-low-risk group with those of the medium-to-high-risk group.

**Table 1:** Characteristics of participants.

Variables	Very-low-to-low risk (n = 185)	Medium-to-high risk (n = 32)	P-value
**Demographics**			
Age (SD)	36.96 (8.09)	44.59 (8.28)	**<0.001**
Sex (%Female)	168 (90.81%)	29 (90.62%)	0.973
Education^b^ (Q1-Q3)	16 (12-16)	14 (12-16)	0.484^a^
**Clinical Characteristics**			
Age at disease onset (Q1-Q3)	28 (22-34)	27.5 (22-34)	0.872^a^
EDSS (Q1-Q3)	1 (1-2.5)	4 (3.25-4.25)	**<0.001** ^**a**^
Disease duration^a^ (Q1-Q3)	8 (4-12)	15 (10.5-20.5)	**<0.001** ^**a**^
**Disease modifying therapy**			
Oral Medications (%)	43 (23.24%)	4 (12.50%)	**<0.001**
Infusion Therapies (%)	58 (31.35%)	23 (71.88%)	
Injectable Therapies (%)	84 (45.41%)	5 (15.62%)	
**Neuropsychological Assessment**			
SDMT (SD)	49.03 (9.86)	35.44 (11.03)	**<0.001**
CVLT-II (SD)	49.22 (9.17)	43.53 (10.22)	**0.002**
PASAT (Q1-Q3)	46 (38-55)	31 (21-44)	**<0.001** ^**a**^
BVMT-R (Q1-Q3)	27 (21-31)	15.5 (9.5-23)	**<0.001** ^**a**^
DAAE score (Q1-Q3)	3 (2-5)	9 (8-10)	**<0.001**

Data are reported as mean (SD), median (Q1-Q3), or frequencies (%).

*: this is the p-value for tests comparing the very-low-to-low and medium-to-high groups.

^a^p-value is from a non-parametric test (Mann-Whitney).

^b^values are presented by years.

BVMT-R: Brief Visuospatial Memory Test-Revised, CVLT-II: California Verbal Learning Test-Second Edition, DMT: disease-modifying therapy, EDSS: Expanded Disability Status Scale, PASAT: Paced Auditory Serial Addition Test, SDMT: Symbol Digit Modalities Test.

Consistent with factors included in the DAAE score, PwMS with medium-to-high risk were older (t(215) = −4.912, p < 0.001), had higher EDSS scores (z = −8.540, p < 0.001), and had longer disease duration (z = −5.408, p < 0.001). Age at onset did not differ significantly between the groups (z = −0.162, p = 0.872). The groups also differed in DMT type: medium-to-high-risk PwMS predominantly received infusion therapies (23/32, 71.88%), whereas very-low-to-low-risk PwMS mainly received injectable therapies (84/185, 45.41%; p < 0.001).

Regarding cognitive performance, very-low-to-low-risk PwMS outperformed medium-to-high-risk PwMS across all domains. SDMT scores were higher (t(215) = 7.074, p < 0.001), as were CVLT-II scores (t(215) = 3.187, p = 0.002). Similarly, PASAT scores differed significantly (z = 4.840, p < 0.001), as did BVMT-R scores (z = 5.045, p < 0.001).

### 3.2. Correlations between risk of conversion and cognitive function

The DAAE score was significantly correlated with all cognitive tests, with the strongest associations observed for SDMT (ρ = −0.484, p < 0.001) and BVMT-R (ρ = −0.392, p < 0.001), followed by PASAT (ρ = −0.338, p < 0.001) and CVLT-II (ρ = −0.267, p < 0.001).

### 3.3. Cognitive tests as predictors of risk of conversion

In total, five models were constructed that predicted the risk of conversion based on patient characteristics and cognitive tests. Four predictive models were constructed with individual cognitive tests, and one was made with the inclusion of all cognitive tests. During the first step of the regression in all models, the usage of infusion therapies and psychiatric comorbidities were revealed as predictive characteristics that are not already accounted for by the DAAE score.

All cognitive domains were independent predictors of the risk of conversion in their respective models. The best-performing models were the SDMT, BVMT-R, PASAT, and CVLT-II models, respectively. When all tests were added during the second step, the R^2^ of the model increased slightly relative to the SDMT model, with SDMT, psychiatric comorbidities, BVMT-R, and infusion therapies retained as significant predictors of the risk of conversion among the cognitive tests (Table 2).

**Table 2:** Hierarchical linear regression models investigating the association between covariates and cognitive domains with risk of conversion.

Cognitive test	Linear Regressions				
	Step 1 (R^2^ = 14.1%):		Coefficient (95%CI)	P-value	Beta
	DMT	Injectable Therapies	Ref		–
		Infusion Therapies	1.216 (0.413 2.020)	**0.003**	0.208
		Oral Medications	−0.045 (−0.986 0.897)	0.926	−0.006
	Psychiatric comorbidities	No	Ref		–
		Yes	3.071 (1.746 4.395)	**<0.001**	0.292
All cognitive tests	Step 2 (R^2^ = 36.0%):				
	DMT	Injectable Therapies	Ref		–
		Infusion Therapies	0.877 (0.175 1.580)	**0.015**	0.150
		Oral Medications	−0.167 (−0.987 0.652)	0.688	−0.024
	Psychiatric comorbidities	No	Ref		–
		Yes	2.329 (1.167 3.500)	**<0.001**	0.221
	SDMT		−0.087 (−0.125 −0.048)	**<0.001**	−0.342
	BVMT-R		−0.062 (−0.117 −0.008)	**0.024**	−0.172
SDMT	Step 2 (R^2^ = 34.4%):				
	DMT	Injectable Therapies	Ref		–
		Infusion Therapies	0.880 (0.171 1.590)	**0.015**	0.150
		Oral Medications	−0.235 (−1.060 0.591)	0.576	−0.034
	Psychiatric comorbidities	No	Ref		–
		Yes	2.378 (1.206 3.551)	**<0.001**	0.226
	SDMT		−0.117 (−0.146 −0.089)	**<0.001**	−0.459
BVMT-R	Step 2 (R^2^ = 29.9%):				
	DMT	Injectable Therapies	Ref		–
		Infusion Therapies	1.008 (0.278 1.738)	**0.007**	0.172
		Oral Medications	−0.001 (−0.854 0.851)	0.998	−0.000
	Psychiatric comorbidities	No	Ref		–
		Yes	2.540 (1.332 3.750)	**<0.001**	0.241
	BVMT-R		−0.146 (−0.187 −0.104)	**<0.001**	−0.403
PASAT	Step 2 (R^2^ = 25.6%):				
	DMT	Injectable Therapies	Ref		–
		Infusion Therapies	1.059 (0.307 1.810)	**0.006**	0.181
		Oral Medications	−0.146 (−1.025 0.733)	0.744	−0.021
	Psychiatric comorbidities	No	Ref		–
		Yes	2.512 (1.261 3.764)	**<0.001**	0.239
	PASAT		−0.076 (−0.102 −0.059)	**<0.001**	−0.344
CVLT-II	Step 2 (R^2^ = 21.3%):				
	DMT	Injectable Therapies	Ref		–
		Infusion Therapies	1.219 (0.448 1.990)	**0.002**	0.208
		Oral Medications	0.072 (−0.833 0.977)	0.875	0.010
	Psychiatric comorbidities	No	Ref		–
		Yes	2.818 (1.542 4.094)	**<0.001**	0.268
	CVLT-II		−0.080 (−0.117 −0.045)	**<0.001**	−0.270

BVMT-R: Brief Visuospatial Memory Test-Revised, CVLT-II: California Verbal Learning Test-Second Edition, DMT: disease-modifying therapy, PASAT: Paced Auditory Serial Addition Test, SDMT: Symbol Digit Modalities Test.

### 3.4. Cognitive tests as predictors of the conversion risk status

In total, five models were constructed that predicted the conversion risk status based on patient characteristics and cognitive tests. Four predictive models utilized individual cognitive tests, while one incorporated all cognitive tests. During the first step of regression in all models, the use of infusion therapies and psychiatric comorbidities emerged as significantly predictive characteristics that were not already accounted for by the DAAE score. With the inclusion of cognitive tests in the second step, these variables remained significant across all five models.

The best-performing models with individual cognitive tests added in the second step were the SDMT model, the BVMT-R model, the PASAT model, and the CVLT-II model, respectively. When all cognitive tests were included in the second step, the model’s R^2^ did not improve upon that of the SDMT model, with SDMT remaining as the only cognitive test independently associated with conversion risk status (Table 3).

**Table 3:** Hierarchical logistic regression models investigating the association between covariates and cognitive domains with conversion risk status.

Cognitive test	Logistic Regressions			
	Step 1 (Pseudo R^2^ = 20.6%):		Odds Ratio (95%CI)	P-value
	DMT	Injectable Therapies	Ref	–
		Infusion Therapies	7.494 (2.461 22.820)	**<0.001**
		Oral Medications	1.908 (0.449 8.115)	0.382
	Psychiatric comorbidities	No	Ref	
		Yes	12.872 (3.894 42.554)	**<0.001**
SDMT	Step 2 (Pseudo R^2^ = 37.7%):			
	DMT	Injectable Therapies	Ref	
		Infusion Therapies	6.137 (1.833 20.542)	**0.003**
		Oral Medications	1.803 (0.402 8.097)	0.442
	Psychiatric comorbidities	No	Ref	
		Yes	11.451 (2.639 49.693)	**0.001**
	SDMT		0.888 (0.846 0.933)	**<0.001**
CVLT-II	Step 2 (Pseudo R^2^ = 25.3%):			
	DMT	Injectable Therapies	Ref	
		Infusion Therapies	8.109 (2.592 25.372)	**<0.001**
		Oral Medications	2.177 (0.510 9.285)	0.293
	Psychiatric comorbidities	No	Ref	
		Yes	12.681 (3.578 44.951)	**<0.001**
	CVLT-II		0.932 (0.887 0.979)	**0.005**
PASAT	Step 2 (Pseudo R^2^ = 31.7%):			
	DMT	Injectable Therapies	Ref	
		Infusion Therapies	6.717 (2.104 21.445)	**0.001**
		Oral Medications	1.794 (0.412 7.817)	0.436
	Psychiatric comorbidities	No	Ref	
		Yes	11.007 (2.895 41.854)	**<0.001**
	PASAT		0.932 (0.901 0.963)	**<0.001**
BVMT-R	Step 2 (Pseudo R^2^ = 34.2%):			
	DMT	Injectable Therapies	Ref	
		Infusion Therapies	7.425 (2.265 24.345)	**0.001**
		Oral Medications	2.329 (0.526 10.307)	0.265
	Psychiatric comorbidities	No	Ref	
		Yes	13.426 (3.224 55.917)	**<0.001**
	BVMT-R		0.869 (0.818 0.924)	**<0.001**
All cognitive tests	Step 2 (Pseudo R^2^ = 37.7%):			
	DMT	Injectable Therapies	Ref	–
		Infusion Therapies	6.137 (1.833 20.542)	**0.003**
		Oral Medications	1.803 (0.402 8.097)	0.442
	Psychiatric comorbidities	No	Ref	
		Yes	11.451 (2.639 49.693)	**0.001**
	SDMT		0.888 (0.846 0.933)	**<0.001**

BVMT-R: Brief Visuospatial Memory Test-Revised, CVLT-II: California Verbal Learning Test-Second Edition, DMT: disease-modifying therapy, OR: odds ratio, PASAT: Paced Auditory Serial Addition Test, SDMT: Symbol Digit Modalities Test.

#### 3.4.1. Effect sizes and power.

Using the Step‑1 model (R² = 0.158) as the reduced specification, the incremental effects in the linear models were: SDMT ΔR² = 0.187 (f²Δ = 0.285), BVMT‑R ΔR² = 0.143 (f²Δ = 0.205), PASAT ΔR² = 0.105 (f²Δ = 0.142), and CVLT‑II ΔR² = 0.061 (f²Δ = 0.078); the combined SDMT+BVMT‑R model yielded ΔR² = 0.202 (f²Δ = 0.316). Post hoc sensitivity analysis indicated ≥0.80 power for all tests when jointly considering the four cognitive predictors in the regression models (≈1.00 for SDMT, BVMT-R, PASAT; ≈ 0.92 for CVLT-II). In logistic analyses, SDMT remained the strongest predictor (OR=0.888, 95% CI 0.846–0.933; pseudo‑R² = 0.377). Full numbers are provided in the Supplementary Material.

#### 3.4.2. Secondary analysis using a Random Forest.

**Fig 1:**
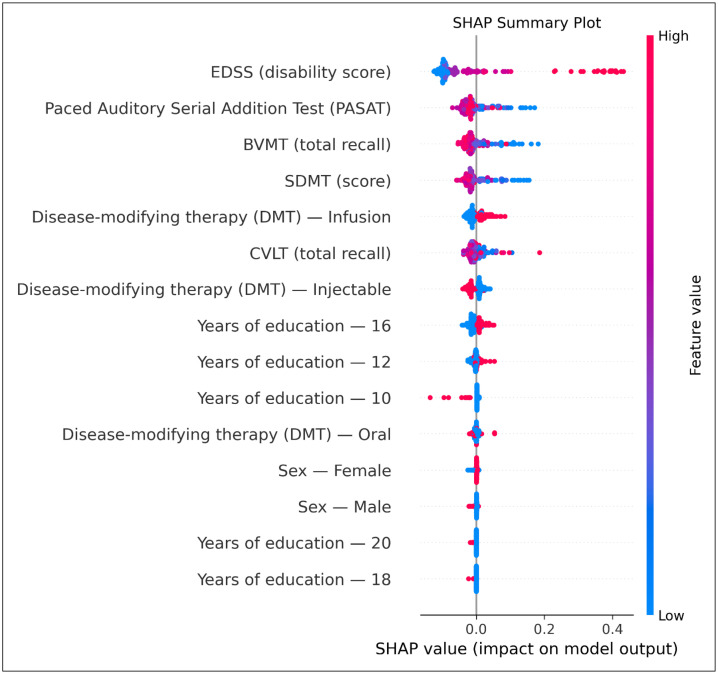
SHAP summary plot illustrating the relative impact of cognitive and clinical variables on Random Forest model predictions of RRMS-to-SPMS conversion risk. Positive SHAP values indicate increased contribution to higher predicted risk. EDSS and cognitive measures (PASAT, BVMT-R, SDMT, CVLT-II) emerged among the strongest predictors.

**Fig 2:**
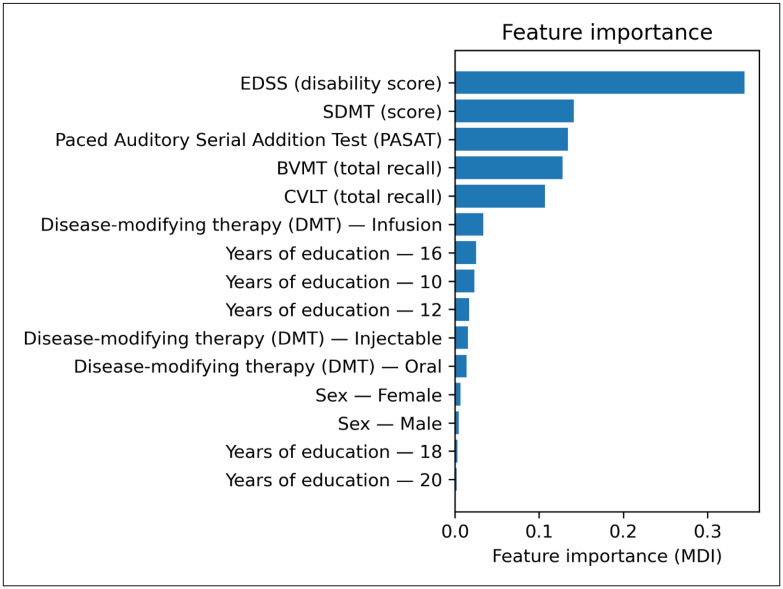
Feature importance (Mean Decrease Impurity, MDI) ranking of cognitive and clinical predictors in the Random Forest model for RRMS-to-SPMS conversion. EDSS contributed the highest importance, followed by SDMT, PASAT, BVMT-R, and CVLT-II, whereas demographic and treatment-related variables showed comparatively lower contributions.

Model evaluation revealed a progressive improvement in discrimination and calibration with the addition of cognitive and EDSS variables. The baseline model demonstrated weak predictive accuracy, characterized by poor diagnostic indices (AUC: 0.59). At the same time, incorporating cognitive tests in Model 1 or EDSS in Model 2 substantially improved accuracy. The full model, which combines cognitive scores and EDSS, achieved the highest predictive accuracy (AUC: 0.98). These results demonstrated that although EDSS values were strongly associated with DAAE Sub scores, the addition of cognitive test scores markedly improved predictive accuracy when combined with baseline variables or EDSS, whereas models relying on cognitive test scores alone showed limited sensitivity (Table 4 and Figs 1 and 2).

**Table 4:** Predictive performance of Random Forest models with different input features for estimating risk of conversion from RRMS to SPMS.

Model	AUC	Brier Score	Sensitivity	Specificity	Accuracy
Baseline	0.594	0.126	0.67	0.63	0.636
Model 1	0.886	0.075	0.67	0.97	0.932
Model 2	0.93	0.07	1	0.84	0.864
Model 3	0.978	0.051	1	0.92	0.932

AUC: area under the curve. Models: Baseline (demographics + DMT type), Model 1 (baseline + cognitive test scores), Model 2 (baseline + EDSS), and Model 3 (baseline + cognitive test scores + EDSS).

AUC: area under the curve, DMT: disease-modifying therapy, EDSS: Expanded Disability Status Scale, RRMS: relapsing-remitting multiple sclerosis, SPMS: secondary progressive multiple sclerosis.

## 4. Discussion

This cross-sectional study examined the correlation and predictive value of cognitive function in relation to the estimated risk of conversion to SPMS in a sample of RRMS patients. While all of the cognitive tests, namely SDMT, CVLT-II, BVMT-R, and PASAT, were associated with the DAAE score, a proxy for risk of conversion, the SDMT, which is an indicator of IPS and sustained attention, was the most strongly correlated with it. Additionally, the models using the SDMT demonstrated the highest accuracy in relation to the estimated risk of conversion to SPMS. Incorporating all cognitive tests into the models resulted in a slight improvement in the association between the DAAE score and the addition of the BVMT-R, an indicator of visuospatial memory. Furthermore, random forest analysis suggested that the association between cognitive performance and SPMS progression is maintained, despite poorer cognitive status, as reflected by a higher EDSS. Despite being among the top variables based on Mean Decrease Impurity (MDI) across input features, CVLT-II had the least impact on final DAAE scores and was overshadowed by the higher relevance of some baseline factors.

Disease progression in MS may occur independently of overt clinical relapses [52] or detectable worsening on conventional clinical [53] or imaging measures [54]. Such progression can occur without clear changes in neurological examination, EDSS scores, or conventional MRI metrics, underscoring the need for complementary markers, including cognitive assessment, to capture early disease worsening. IPS, whether visual (assessed by SDMT) or auditory (assessed by PASAT), was associated with the DAAE score. Integrating cognitive assessments with EDSS and DAAE can enhance their accuracy by identifying cognitive changes associated with relapse activity [55]. Considering that cognitive decline in RRMS may occur insidiously beyond clinically documented relapses and physical impairments [17], the incorporation of cognitive Progression Independent of Relapse Activity into DAAE may be beneficial. Our results regarding SDMT are in agreement with the literature, which has identified SDMT as a promising biomarker of conversion to SPMS [56]. Although generally regarded as a sensitive test and interpreted as a measure of IPS, the SDMT also engages multiple processes, including memory and lexical access speed. Therefore, it serves as a general measure of cognition rather than a specific test of IPS [57]. While this makes the SDMT suitable for screening cognitive impairment, it may fail to capture the specific cognitive domains most affected in PwMS at high risk of conversion to SPMS. As a result, more specific tests should be used in future research to identify the affected domains.

In addition, we found that verbal learning and memory (assessed by the CVLT-II) and visuospatial memory (assessed by the BVMT-R) were associated with DAAE, albeit to a lesser extent. Information on these tests and SPMS is sparse in the literature; as a result, a direct comparison is not possible. However, poorer CVLT-II and BVMT-R performance has been associated with reduced mean cortical thickness [58]. Notably, cortical atrophy progresses more rapidly in SPMS relative to RRMS and clinically isolated syndrome [59,60]. This suggests that PwMS with poorer test results may experience a more rapid rate of cortical atrophy and, therefore, may be at a greater risk of conversion to SPMS, offering a plausible biological context for our results. Importantly, brain atrophy was not directly assessed in the present study, and this interpretation should therefore be considered exploratory. PASAT was also associated with DAAE. However, its association with conversion to SPMS remains understudied. In a study on 2,355 RRMS patients, PASAT was a predictor of conversion in univariate analyses, yet lost its significance in multivariate analyses [61]. Interestingly, the timed 25-foot walk test was a significant predictor of conversion, consistent with prior associations with cognitive function [62] and conversion [63]. Other predictors of conversion include iron rim lesions [63] and depression [63], both of which have been associated with cognitive function [64,65]. Overall cognitive function, especially SDMT, can serve as a biomarker for early detection of MS progression. Other biomarkers found in our study included psychiatric comorbidities and the type of DMTs.

There is no strong evidence for the psychiatric comorbidities predicting conversion to SPMS, yet it has been associated with disability progression [66] and progressive forms of MS have slightly higher prevalence rates for psychiatric comorbidities compared to RRMS [67]. While more studies are required before a definitive conclusion can be made, early screening and management of psychiatric comorbidities may help prevent conversion to SPMS. Our finding of infusion DMTs reducing the risk of conversion is also supported by the literature and may help clinical decision makers regarding DMT selection for RRMS patients [68].

### 4.1. Limitations

There are some caveats when interpreting the results of our study. While DAAE has been shown to be a useful measure for predicting risk of conversion, it is far from perfect. One component is disease duration, defined as the time since diagnosis rather than since symptom onset. This may introduce bias into the measure, especially in populations of PwMS who are less likely or unable to seek medical attention, such as men [69] or impoverished communities [70]. Second, the EDSS, as a component of the DAAE score, has notable limitations, and its calculation can vary considerably depending on the examiner [71]. More objective, examiner-independent measures, such as the T25FW and 9-HPT, may be better suited for predicting the risk of conversion [72,73]. In our study, the correlation between EDSS and cognitive function may also introduce a confounding factor. Furthermore, disease severity at onset is a recognized prognostic factor for conversion, not captured by the DAAE score, and could be valuable in future research [74]. Additionally, although we measured three cognitive domains, other cognitive domains were not explored. In this regard, visual evoked potential latency may be a useful measure in future studies, as it correlates with the number of impaired cognitive domains, rather than specific domains [75]. Lastly, cognition is not a comprehensive measure of disease progression and should be used in conjunction with MRI findings, disability progression, and clinical relapses, which are commonly incorporated into No Evidence of Disease Activity [76]. This enables us to quantify and characterize disease progression in a multidimensional, examiner-independent manner.

The DAAE score is an estimate of the risk of conversion, and the actual risk of conversion (i.e., the ratio of PwMS who convert from RRMS to SPMS over a given period) may vary from this estimate. In addition, the cross-sectional design limits the ability to directly assess conversion risk, which is inherently a longitudinal concept. As the DAAE score estimates the 5-year risk of conversion from RRMS to SPMS [32], a cohort study, ideally spanning at least five years, would provide a more accurate representation of the progression of cognitive impairment leading to SPMS. Moreover, we were able to assess only the associations of four cognitive tests in this study; other cognitive domains remain unexplored. Future studies should examine other neuropsychological assessments to provide a more precise understanding of how cognition relates to disease progression. Additionally, unmeasured confounders, such as comorbid conditions and lifestyle differences, may have influenced the associations observed in this study. Post hoc sensitivity and power estimates were based on incremental effect sizes (f²Δ) in linear models; power approximations for logistic models, which rely on pseudo‑R², are inherently approximate and should be interpreted with caution. The close association between EDSS and cognitive performance may still introduce residual bias; however, our additional Random Forest analyses suggest that the predictive role of cognition is not fully attributable to this overlap. Moreover, although age was inherently incorporated into the DAAE score, it was not included as an independent covariate to avoid collinearity. Therefore, subtle age-related effects on cognitive performance may not have been eliminated. Furthermore, although data on the type of DMTs were available, we were unable to include additional treatment-related variables such as the number of disease exacerbations, duration of therapy, and treatment changes over time, as these data were not consistently available in our cross-sectional dataset. These factors may significantly influence cognitive decline and disease progression, and future longitudinal studies should incorporate them to provide a more comprehensive assessment.

## 5. Conclusion

In conclusion, this study highlights the independent association between cognitive function and the risk of conversion from RRMS to SPMS. While the BVMT-R, CVLT-II, and PASAT were also associated with the DAAE score, the SDMT emerged as the strongest predictor of both conversion risk and conversion risk status. These findings suggest that cognitive deterioration is not only a feature of SPMS but may also be a risk factor for the disease. We recommend that future longitudinal studies investigate this relationship while accounting for a wide range of confounders to draw more reliable conclusions.

## Supporting information

S1 File Effect sizes in linear and logistic regressions. (XLSX)
